# Neurofibromatosis of the nipple-areolar area: a case series

**DOI:** 10.1186/1752-1947-4-22

**Published:** 2010-01-25

**Authors:** Maria Rita Bongiorno, Spyridoula Doukaki, Mario Aricò

**Affiliations:** 1 Department of Dermatology, University of Palermo, Sicily, Palermo, Italy

## Abstract

**Introduction:**

Neurofibromatosis type 1 is an autosomal dominant disorder that occurs across all ethnic groups and affects approximately one in 4000 individuals. One of the most noticeable characteristics of the disease is the development of neurofibromas.

**Case presentation:**

A total of 258 patients (131 women, 127 men) with neurofibromatosis type 1 were evaluated between 1994 and 2004 in our hospital's dermatology department. Nine patients (3.45%, 95% confidence limits 1.22 to 5.68) had neurofibromas of the breast. One of these nine patients presented with an extensive congenital plexiform neurofibroma in the outer quadrants of her right breast, extending to the nipple-areolar complex. Meanwhile, three patients had more than one neurofibroma on the nipple-areolar complexes. Three patients had a family history of neurofibroma. Over the years 1994 to 2004, the cutaneous lesions were not associated with any malignancies. Presenting symptoms were related to conditions such as increasing size of the mass, and associated loss of function and pain.

**Conclusions:**

This study suggests that the changes are limited to particular subgroups. That neurofibromatosis is more prevalent in women (7 women and 2 men) suggests that being female could be a susceptibility factor for the development of neurofibromas of the nipple-areolar complexes. There are few reports in the literature describing breast carcinomas in association with von Recklinghausen disease. It has been speculated that the presence of multiple neurofibromas of the breast may obscure a breast mass at palpation, leading to a delay in clinical detection. We suggest that patients with neurofibromas of the breast have more rigorous clinical and mammographic screening of the breast during adulthood to determine the presence or absence of malignancies. The finding that both the neurofibromatosis type 1 gene and a breast cancer predisposition gene are located in close proximity on chromosome 17q makes the association of these two conditions intriguing.

## Introduction

Neurofibromatosis type 1 (NF1) is one of the most common autosomal dominant disorders as it affects approximately 1 in 4000 individuals [1]. It is associated with deletions, insertions or mutations in the NF1 gene, witch is a tumour suppressor gene located in the pericentromeric region of chromosome 17. A substantial body of evidence supports the hypothesis that neurofibromin, the NF1 gene product, has a role in cell growth and differentiation [1].

NF1 is characterized by a variety of benign and malignant lesions, such as multiple café-au-lait spots, inguinal and axillary freckling, cutaneous neurofibromas, plexiform neurofibromas, optic nerve gliomas, skeletal abnormalities, phaeochromocytomas, and malignant peripheral nerve sheath tumours. The morbidity and mortality caused by NF1 are dictated by the occurrence of complications that involve any of the body systems. Manifestations of NF1 vary at different times in an individual's life. Substantial variability also exists among affected members of a single family. At least half of the patients with NF1 will have only cutaneous involvement that can be a source of psychological burden as a result of cosmetic disfigurement [2].

One of the most noticeable characteristics of the disease is the development of neurofibromas, especially on the trunk and limbs. Four clinically and morphologically distinct variants of neurofibromas occur in neurofibromatosis 1: cutaneous lesions, localized intraneural tumours, plexiform neurofibromas, and massive soft tissue neurofibromas. Cutaneous neurofibromas present as sessile and dome-shaped, sometimes pedunculated, flesh-coloured, and with soft papules or nodules. Patients with cutaneous neurofibromas are usually asymptomatic, but they can be pruritic. On the other hand, subcutaneous neurofibromas are usually larger than dermal lesions and consist of fusiform swelling that occurs along the sheaths of peripheral nerves. They do not infiltrate surrounding tissues but can grow to an enormous size.

About 95% of patients have discrete benign neurofibromas. These lesions do not usually develop before adolescence, may be quite variable in size, and may increase in number, as the patient grows older. The plexiform variant of neurofibromas involves single or multiple nerve fascicles that often arise from the branches of major nerves and form a mass of tangled, rope-like structures that feel similar to a "bag of worms" on palpation and can be associated with massive soft-tissue overgrowth, leading thus to functional impairment. Most plexiform neurofibromas are present at birth or become apparent during the first years of a life in 30% of patients diagnosed with neurofibromatosis type 1 [3].

A total of 258 patients (131 women, 127 men) with neurofibromatosis type 1 were evaluated between 1994 and 2004 in our hospital's Dermatology Department. All patients included in this study have NF1 as defined by the National Institute of Health Consensus Conference [4,5]. We excluded cases described as segmental NF or those which were classified as "other type of NF".

In this report we present 9 patients with neurofibromas of the nipple-areolar complex.

## Case presentation

A systematic multidisciplinary clinical investigation and familial enquiry were performed for each patient (Table 1).

**Table 1:** Nine cases of patients with NF1 presenting with neurofibromas of the breast protruding from the nipple-areolar complexes.

Case no	Age	Sex	Family history	Histopathology of neurofibromas of the nipple-areolar complexes	Plexiform neurofibromas	Axillary freckling	Café au lait spots	Cutaneous neurofibromas	Coexisting disease
1	44	M	No	Neurofibroma		No	Yes	Yes	

2	66	M	Yes	Neurofibroma		Yes	Yes	Yes	

3	32	F	No	Neurofibroma		No	Yes	Yes	

4	50	F	No	Neurofibroma		Yes	Yes	Yes	

5	70	F	Yes	Neurofibroma		No	Yes	Yes	Scoliosis

6	51	F	Yes	Neurofibroma		No	Yes	Yes	

7	74	F	No	Neurofibroma		Yes	Yes	Yes	

8	40	F	No	Neurofibroma	Yes	Yes	Yes	Yes	

9	35	F	No	Plexiform neurofibroma	Yes	No	Yes	Yes	Vitiligo

### Case report 1

A 44-year-old man presented with multiple café-au-lait spots and neurofibromas. Upon inspection of his chest and breasts, a cutaneous neurofibroma was noted on his left nipple-areolar complex. He had no family history of neurofibromatosis.

### Case report 2

The patient was a 66-year-old man with neurofibromatosis type 1. His mother and all his siblings had neurofibromatosis 1. Clinical examination showed that he had café-au-lait spots and multiple neurofibromas in a generalized distribution. Moreover, a large subcutaneous neurofibroma of approximately 8 cm in diameter was palpable on his occipital region. A neurofibroma was also noted on his right nipple.

### Case report 3

The patient was a 32-year-old woman with few scattered neurofibromas and café-au-lait spots. She had no family history of neurofibromatosis. Examination revealed a neurofibroma of 10 mm in diameter involving the cutaneous and subcutaneous tissues involving her left breast to the areola. Two small neurofibromas were also noted in close proximity to her left nipple.

### Case report 4

**Figure 1:**
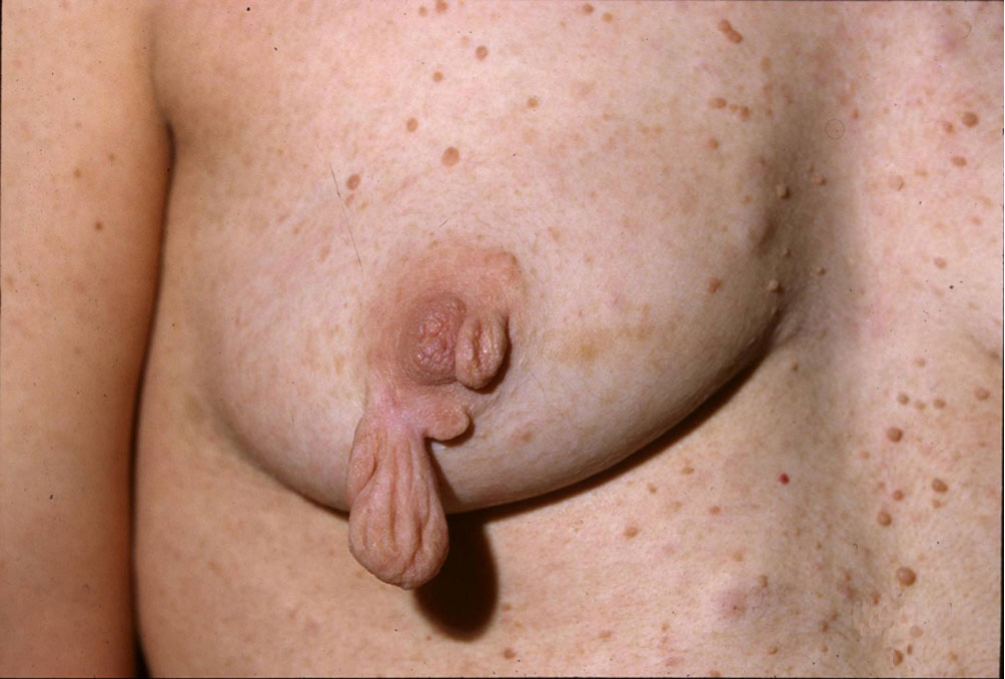
**Case 4**. The lesions are large and pedunculated protruding 2 cm and 6 cm, respectively, from the nipple-areolar complexes.

A 50-year-old woman was referred to our hospital for evaluation of multiple neurofibromas on her trunk, head and neck. She also had several café-au-lait spots on her axilla. She had no family history of neurofibromatosis. Physical examination showed two neurofibromas on the right nipple-areolar complex of this patient. The lesions were large and pedunculated, and extended outward 2 cm and 6 cm respectively from the areolar region (Figure 1). These neurofibromas were seen to increase in size over a 4-year period. Mammography showed dense cutaneous well-circumscribed pedunculated nodules arising from the areolar region.

### Case report 5

The patient was a 70-year-old woman who presented with large neurofibromas, café-au-lait spots and scoliosis. She reported that her mother had neurofibromatosis type 1. Upon examination of her breasts a neurofibroma on the left nipple-areolar complex was noted.

### Case report 6

**Figure 2:**
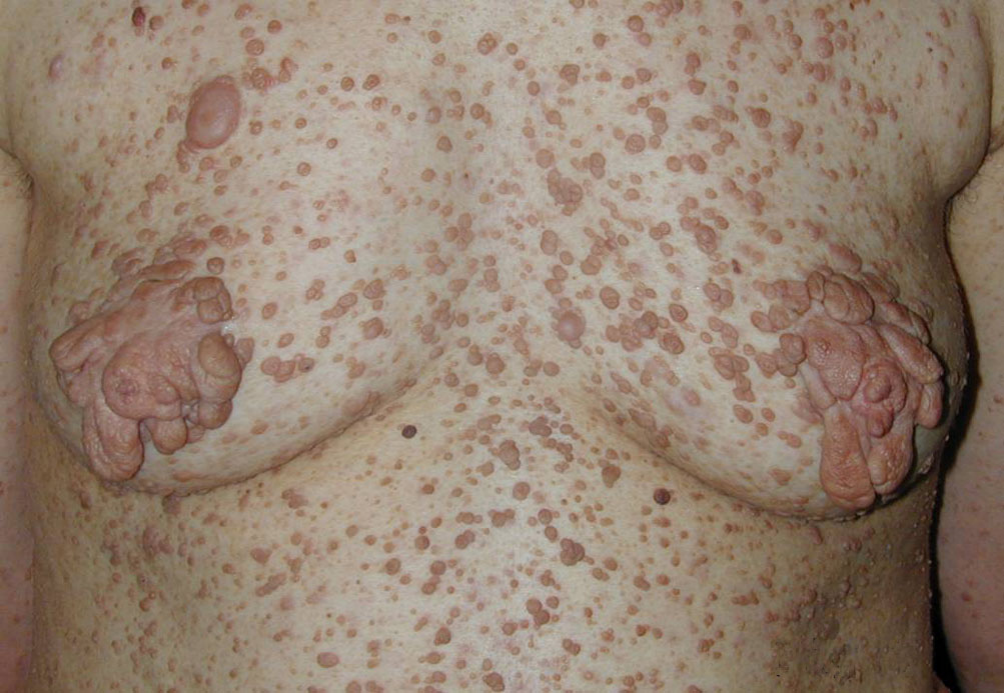
**Case 6**. The lesions deforming both nipples and areolar regions.

A 51-year-old woman presented with numerous neurofibromas, several café au lait spots and NF1 family antecedents. On examination, multiple cutaneous neurofibromas were noted on her chest and breasts. In particular, bilateral large serpiginous, pedunculated neurofibromas were prominent. The lesions were painful and extended outward at least 7 cm to 8 cm from the nipple and areola, thus deforming both nipples (Figure 2). Mammography showed bilateral dense breasts, as well as multiple, cutaneous, well-circumscribed, pedunculated nodules arising from both nipple-areolar regions.

### Case report 7

The patient was a 74-year-old woman with no family history of neurofibromatosis. On examination, there was noted skin involvement with numerous patches of cutaneous pigmentation and extensive cutaneous tumours. The hue abnormalities were light to dark brown and diffuse all over the skin. The neurofibromas were soft, flesh coloured, and non-painful tumours that ranged in size from several millimetres to many centimetres in diameter. Moreover, the patient had a neurofibroma projecting over the left nipple-areolar complex, which markedly deformed her nipple.

### Case report 8

A 40-year-old woman without family history of neurofibromatosis presented to our department with café-au-lait spots spread all over her body. The café-au-lait macules were flat, light to dark brown and well-circumscribed areas that range from a few millimetres to several centimetres in diameter. Intertriginous freckling, discrete cutaneous neurofibromas, and diffuse subcutaneous neurofibromas were also observed. Upon chest and breast examination, a neurofibroma close to her right nipple and a nodular plexiform neurofibroma on the chest's right anterior region were found. The nodule was tender and firm along the nerve plexuses.

### Case report 9

**Figure 3:**
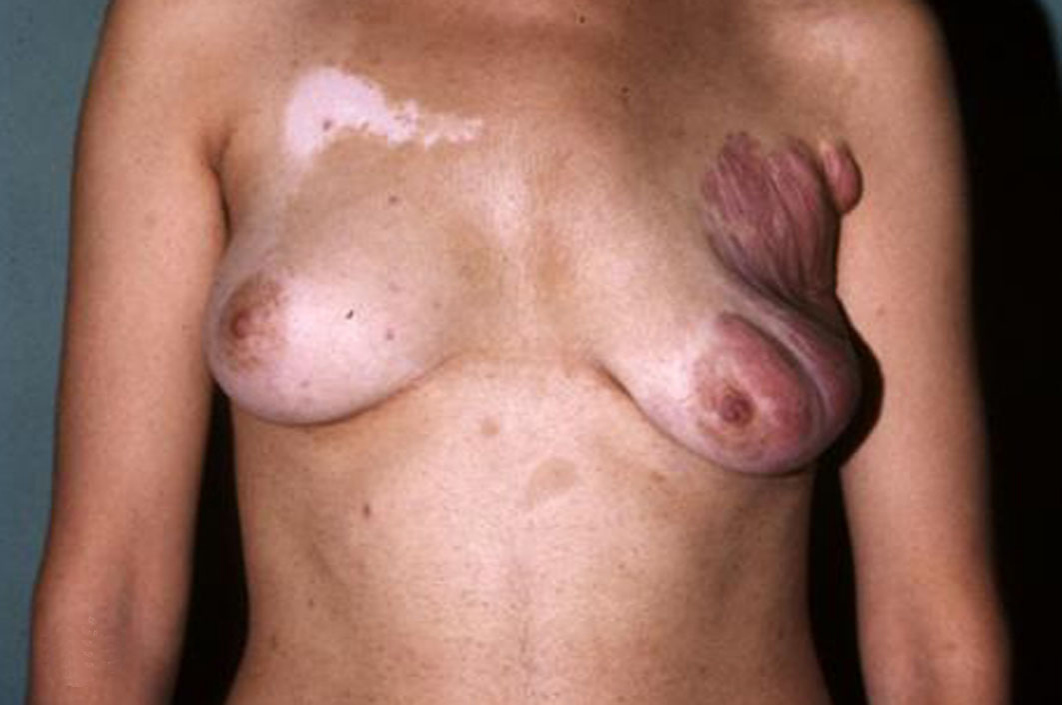
**Case 9**. The extensive congenital plexiform neurofibroma involved the outer quadrants of her right breast and extends to the nipple-areolar complex and to the homolateral axillary region and arm.

The patient was a 35-year-old woman with no family history of neurofibromatosis. An inspection of her skin revealed extensive congenital plexiform neurofibroma in the outer quadrants of her right breast that extended to the nipple-areolar complex and to the homolateral axillary region and arm (Figure 3). The lesion infiltrates the nerve itself and the surrounding tissues, which was leading to soft tissue overgrowth and causing dysfunction and disfigurement. Dermatological exploration also showed some smooth, margined, light-brown pigmented macules on her chest that varied in size and configuration. Moreover, a segmental vitiligo characterized by unilateral macules in dermatomal distribution was present on her left lower limb.

**Figure 4:**
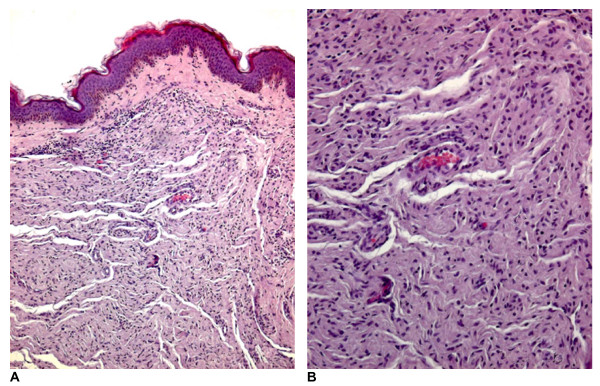
**Case 4**. The neurofibroma is composed of widely spaced cells with elongated nuclei and scant cytoplasm and embedded in mucopolysaccaride-rich, variably collagenous matrix (hematoxylin and eosin staining, original magnification (A) ×125, and (B) ×250).

Microscopic findings of biopsy specimens of the lesions on the nipple-areolar complexes were obtained from all patients to confirm the diagnosis on histological grounds. Hematoxylin and eosin stained sections from sections that were fixed with formalin and embedded in paraffin were prepared. On histology, the nipple lesions of patients 1 to 8 were identified as neurofibromas. The neurofibromas contained interlacing bundles of elongated cells with dark staining nuclei. The cells were associated with strands of collagen, and small to moderate amounts of mucoid material separated the cells from the collagen. Occasional mast cells and lymphocytes were also present in the stroma. In particular, the 4^th ^and 6^th ^patients had neurofibromas that were composed of widely spaced cells devoid with elongated nuclei and scant cytoplasm and embedded in matrices that were rich in mucopolysaccharide and variably collagenous (Figures 4a and 4b). The collagen fibres were typically delicate and lay within a matrix that was variable, abundant and rich in mucopolysaccharide.

**Figure 5:**
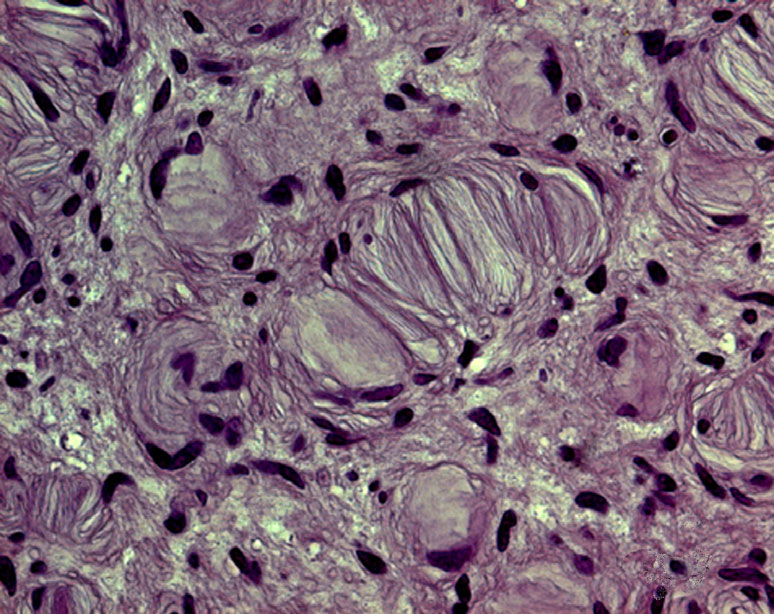
**Case 9**. The cell formations resemble Wagner-Meissner corpuscles. These are spherical and aggregated (haematoxylin and eosin staining; original magnification ×640).

Histological finding showed plexiform neurofibroma in the 9^th ^patient. The neurofibroma is located in both the dermal and subcutaneous tissues. The neurofibroma cells surround the adipose tissue. Tactile differentiation was apparent, and the pseudomeissnerian corpuscles were found to be spherical and aggregated (Figure 5).

In all the patients we describe, clinical and histopathological examination at the time of their first presentation, as well as their subsequent examinations, did not show an association with breast carcinoma.

## Discussion

Nine out of 258 patients, or 3.45% of the total number (95% confidence limits: 1.22 to 5.68), of the patients evaluated with a diagnosis of NF1 in our dermatology department harboured neurofibromas of the breast. Of this number, one patient presented with an extensive congenital plexiform neurofibroma in the outer quadrants of the right breast that extended to the nipple-areolar complex, and 3 patients harboured more than one neurofibroma on the nipple-areolar complexes. With 127 male patients and 131 female patients studied, the gender distribution of the cohort was comparable. The most striking and singular observation of this study was the gender difference. We found a significant number of women (7 patients) with neurofibromas of the nipple-areolar complexes. A family history was reported in 3 patients. Over the years, the cutaneous lesions were not associated with malignancies.

NF1, known also as Von Recklinghausen disease, is an autosomal dominant hamartomatous disease primarily involving the neuroectodermal and mesodermal tissues. Although the clinical manifestations of NF1 are well known [6], the course of the condition in individual patients is largely unpredictable. This unpredictability and the general progression of the disease is a major concern for most patients with NF1 and their families [7].

NF1 primarily affects the peripheral nervous system and is often characterized by large numbers of neurofibromas.

Neurofibromas of the breast are quite rare manifestations of patients with NF1. In such cases, they occur on the nipple-areolar complexes [8,9], and their frequency increases with age. Reviewing the literature, several clinic-based series of patients with NF1 have been reported, but only a few reports have specifically examined neurofibromas of the nipple-areolar complexes [10,11]. In this study, we present information about the clinical features of neurofibromas in patients with NF1, with specific regard to the location of tumours and presenting symptoms. To the best of our knowledge, this series is the largest investigation of patients with neurofibromas of the nipple-areolar complexes and NF1 to date. Previous studies do not provide information on gender, or include fewer patients [8-11].

In our patients, neurofibromas of the nipple-areolar complexes were generally soft, flattened, or pedunculated skin lesions that protrude from the nipple-areolar regions and eventually deformed the nipples. The presenting symptoms were related to increasing size of the mass, associated loss of function and the feeling of pain. On mammography, the typical appearance was of a single or multiple skin lesions projecting over the mammary parenchyma. Portions of the lesions were outlined by air, demonstrating well-defined smooth margins [10].

The observation of a female predominance in our group suggests that female gender could be a susceptibility factor for the development of neurofibromas of the nipple-areolar complexes. To estimate the real frequency of the neurofibromas of the nipple-areolar complexes and to determine whether there is a familial tendency, a detailed family history and complete physical examination of affected patients and family members are warranted.

This study suggests that such changes are limited to particular subgroups. The mechanisms by which mutations of the NF1 gene produce these phenotypic effects are unknown, but understanding how they do so may provide an important clue to the pathogenesis of the more serious manifestations of NF1.

The phenotype of NF1 is highly variable, and some affected individuals are more likely than others to develop certain features of the disease. Although various abnormalities, including chromosome rearrangements, deletions, insertions, duplications, and base substitutions have been reported, the wide diversity of mutation types and wide range of variable expression of neurofibromatosis 1 have made it difficult to establish genotype to phenotype correlations. The one exception to the lack of genotype to phenotype correlation is the case of entire gene deletions that appears to be associated with the early onset of a large number of cutaneous neurofibromas, minor facial anomalies, and developmental delay [12].

Certain clinical features of NF1 share a common pathogenesis, while other features develop through different pathogenic mechanisms. Further clinical, epidemiological, pathological, and molecular studies are necessary to elucidate the basis for these associations in patients with NF1.

NF1 also represents a major risk factor in the development of several malignancies, particularly malignant peripheral nerve sheath tumours (MPNST) [13], optic gliomas, other gliomas, rhabdomyosarcoma, astrocytoma and neurofibrosarcoma and leukemias. The average life expectancy of patients with NF1 is probably reduced by 10 to 15 years, and malignancy is the most common cause of death [11]. In addition, there are few cases in the literature describing invasive ductal carcinomas in association with von Recklinghausen disease [10]. Breast cancer has a lifetime incidence of up to one in eight women. Owing to the paucity of reports of NF1 and breast cancer, Riccardi commented that an association between these two types of diseases cannot be firmly established, but recommended molecular analysis of breast cancers in NF1 patients [14]. The finding that both the NF1 gene and a breast cancer predisposition gene (BRCA1) are located in close proximity on chromosome 17q makes the association of these two conditions intriguing.

It has been speculated that the presence of multiple neurofibromas of the breast, which can develop both on the surface of the skin and subcutaneously, may obscure a breast mass at palpation, leading thus to a delay in clinical detection [10]. Patients usually do not seek medical assistance because they suppose that it is simply a presentation of their known von Recklinghausen disease. If a suspected breast lesion is demonstrated in a patient with NF1, radiological imaging of the mass is recommended in order to obtain further diagnostic information. Careful mammographic interpretation in these patients is also important [10].

## Conclusions

This report aims to stimulate interest in the unusual presentation of neurofibromas on the nipple-areolar complexes. It is hoped that clinicians will become aware that breast cancer can be difficult to detect in these patients, leading thus to a delay in clinical detection. We suggest that patients with neurofibromas of the breast have more rigorous clinic and mammographic screening of the breast during adulthood to determine the presence or absence of malignancies. The finding that both the NF1 gene and a breast cancer predisposition gene (BRCA1) are located in close proximity on chromosome 17q makes the association of these two conditions intriguing [15], even if the risk of developing malignant transformation in neurofibromas of the nipple-areolar complexes is rare.

## Abbreviations

BRCA1: breast cancer 1; NF1: neurofibromatosis type 1; MPNST: malignant peripheral nerve sheath tumours.

## Consent

Written informed consent was obtained from the patients for publication of this case series and any accompanying images. A copy of the written consent is available for review by the Editor-in-Chief of this journal.

## Competing interests

The authors declare that they have no competing interests.

## Authors' contributions

BMR analyzed and interpreted the patient data and was a major contributor in writing the manuscript. DS analyzed and interpreted the patient data and was a major contributor in writing the manuscript. AM analyzed the data and was involved in drafting the manuscript and revising it for important critical content. All authors read and approved the final manuscript.

## References

[B1] Nussbaum RL McInnes RR Willard HF Genetics and cancer Genetics in Medicine 2001 Philadelphia: WB Saunders Company

[B2] Wolkenstein P Zeller J Revuz J Ecosse E Leplège A Quality of life impairment in neurofibromatosis type 1: a cross-sectional study of 128 cases Arch Dermatol 2001 137 1421 1425 11708944 10.1001/archderm.137.11.1421

[B3] Ward BA Gutmann DH Neurofibromatosis 1: from lab bench to clinic Pediart Neurol 2005 32 221 228 10.1016/j.pediatrneurol.2004.11.002 15797177

[B4] NIH Consensus Development Conference Neurofibromatosis Arch Neurol 1988 45 575 578 3128965

[B5] Gutmann DH Aylsworth A Carey JC Korf B Marks J Pyretiz RE Rubenstein A Viskochil D The diagnostic evaluation and multidisciplinary management of neurofibromatosis 1 and neurofibromatosis 2 JAMA 1997 278 51 57 10.1001/jama.278.1.51 9207339

[B6] Riccardi VM Neurofibromatosis: Phenotype, Natural History and Pathogenesis 1992 2 Baltimore: Johns Hopkins University Press

[B7] Friedman JM Birch PH Type 1 neurofibromatosis: a descriptive analysis of the disorder in 1,728 patients Am J Med Genet 1997 70 138 143 10.1002/(SICI)1096-8628(19970516)70:2<138::AID-AJMG7>3.0.CO;2-U 9128932

[B8] Sherman JE Smith JW Neurofibromas of the breast and nipple-areola area Ann Plast Surg 1981 7 302 307 10.1097/00000637-198110000-00010 6797340

[B9] Fink D Schneider C Wight E Perucchini D Haller U Neurofibromatosis of the breast in a patient with morbus von Recklinghausen Gynakol Geburtshilfliche Rundsch 2000 40 47 49 10.1159/000022328 10867496

[B10] Millman SL Mercado CL An unusual presentation of neurofibromatosis of the breast Breast J 2004 10 1 45 47 10.1111/j.1524-4741.2004.09616.x 14717759

[B11] Murata A Kansizb F Kabakusc N Kazez A Ozercan R Neurofibroma of the breast in a boy with neurofibromatosis type 1 Clin Imaging 2004 28 6 415 417 10.1016/S0899-7071(04)00004-X 15531141

[B12] Tonsgard JH Yelavarthi KK Cushner S Short MP Lindgren V Do NF1 gene deletions result in a characteristic phenotype? Am J Med Gent 1997 73 80 86 10.1002/(SICI)1096-8628(19971128)73:1<80::AID-AJMG16>3.0.CO;2-N 9375928

[B13] Bilgic B Ates LE Demiryont M Ozger H Dizdar Y Malignant peripheral nerve sheath tumors associated with neurofibromatosis type 1 Pathol Oncol Res 2003 9 201 205 10.1007/BF03033740 14530818

[B14] Teh BT Birrell G Farrell A Leonard JH Walters MK Palmer JM Ramsay JR Schlect DJ Furnival C Lavin MF Bennett I Hayward NK Breast cancer in six women with neurofibromatosis type 1 The Breast 1997 6 155 160 10.1016/S0960-9776(97)90558-0

[B15] Ceccaroni M Genuardi M Legge F Lucci-Cordisco E Carrara S D'Amico F Greggi S Scambia G BRCA1-related malignancies in a family presenting with von Recklinghausen's disease Gynecol Oncol 2002 86 375 378 10.1006/gyno.2002.6757 12217765

